# Carbapenemase-Producing Gram-Negative Bacteria in Andalusia, Spain, 2014–2018

**DOI:** 10.3201/eid2609.191772

**Published:** 2020-09

**Authors:** Inmaculada López-Hernández, Mercedes Delgado-Valverde, Felipe Fernández-Cuenca, Lorena López-Cerero, Jesús Machuca, Álvaro Pascual

**Affiliations:** Laboratorio PIRASOA, Hospital Universitario Virgen Macarena, Seville, Spain (I. Lopez-Hernandez, M. Delgado-Valverde, F. Fernández-Cuenca, L. López-Cerero, J. Machuca, Á. Pascual);; Instituto de Biomedicina de Sevilla IBIS, Hospital Universitario Virgen Macarena, Consejo Superior de Investigaciones Científicas, Universidad de Sevilla, Seville (I. López-Hernández, M. Delgado-Valverde, F. Fernández-Cuenca, L. López-Cerero, J. Machuca, Á. Pascual);; Red Española de Investigación en Patología Infecciosa (REIPI RD16/0016), Instituto de Salud Carlos III, Madrid, Spain (I. López-Hernández, M. Delgado-Valverde, F. Fernández-Cuenca, L. López-Cerero, J. Machuca, Á. Pascual);; Universidad de Sevilla, Seville (Á. Pascual).

**Keywords:** *Acinetobacter baumannii*, AMR, antimicrobial resistance, bacteria, carbapenemases, *Citrobacter freundii*, cloxacillin, dipicolinic acid, *Enterobacter cloacae*, *Escherichia coli*, gram-negative bacteria, *Klebsiella oxytoca*, *Klebsiella pneumoniae*, molecular epidemiology, phenyl boronic acid, PIRASOA, *Pseudomonas aeruginosa*, Spain

## Abstract

The emergence and spread of carbapenemase-producing gram-negative bacteria is a major public health concern. We used data collected from microbiology laboratories as part of the PIRASOA program during 2014–2018 to study the epidemiology of carbapenemase-producing bacteria in Andalusia, Spain. Our findings highlight the importance of ongoing surveillance and epidemiologic studies for these bacteria.

There are 3 common carbapenemase classes: class A includes *Klebsiella pneumoniae* carbapenemase (KPC); class B is metallo-β-lactamases (MBL), including Verona integron-encoded metallo-β-lactamase (VIM), New Delhi metallo- β-lactamase (NDM) and imipenemase (IMP); and class D includes oxacillinases (OXA), including OXA-48–like carbapenemases. In Spain, all 3 of these classes have been reported in recent studies (*1*–*4*). A multicenter study of *Enterobacterales* carbapenemase-producing gram-negative bacteria (CPGNB), isolated from urine specimens, found a prevalence of 1.6%. OXA-48 was the most prevalent, followed by KPC and MBL (*5*). 

In Andalusia, a region in the south of Spain with a population of ≈8.4 million people, The PIRASOA (https://www.iavante.es/es/programa-pirasoa) program was designed to prevent and control healthcare-associated infections and promote the appropriate use of antimicrobials, covering the entire area of the Andalusia public healthcare system, which includes 34 hospitals in 8 provinces. Data collected through PIRASOA during 2014–2018 provided an opportunity for us to study and understand the epidemiology of carbapenemase-producing bacteria in Andalusia. 

## The Study

During January 2014–December 2018, microbiology laboratories in the hospitals included in the PIRASOA program voluntarily submitted CPGNB detected according to EUCAST screening criteria (*6*) to the PIRASOA reference laboratory. We identified the isolates using MALDI-TOF Biotyper (Bruker Daltonics, https://www.bruker.com). Antimicrobial susceptibility testing was performed by microdilution (Microscan; Beckman Coulter, https://www.beckmancoulter.com) and interpreted according to EUCAST breakpoints (*7*). We determined the inhibition of carbapenemase activity by using inhibitors (dipicolinic acid, phenyl-boronic acid, and cloxacillin). In addition, we tested all the isolates with the β-CARBA test (Bio-Rad, https://www.bio-rad.com) and, from January 2018, also with the NG-test CARBA 5 (NG Biotech, https://ngbiotech.com). All isolates with a positive or indeterminate result from phenotypic screening were tested by PCR and sequencing (Macrogen, https://dna.macrogen.com) (*bla*_KPC_, *bla*_OXA-48_, *bla*_VIM_, *bla*_IMP_, *bla*_NDM_, *bla*_OXA-23,24/40,58_) or by whole genome sequencing on a MiSeq system (Illumina, https://www.illumina.com), which became available in October 2017. We evaluated genetic relatedness by pulsed-field gel electrophoresis (CHEF DR-II system; Bio-Rad); isolates of <2 bands difference were assigned to the same cluster. We determined multilocus sequence typing using PCR and sequencing or whole-genome sequencing data (https://cge.cbs.dtu.dk/services/MLST). 

During the study period, we analyzed 2,005 gram-negative isolates of which 1,243 (62%) were carbapenemase producers. Among those 1,243 isolates, clinical specimens (64%, 791/1,243) comprised the predominant source, followed by surveillance (31%, 387/1,243) and environmental samples (5%, 65/1,243). The most common species were *K. pneumoniae* (45%, 560/1,243), *Acinetobacter baumannii* (34%, 425/1,243), and *K. oxytoca* (7%, 83/1,243). 

KPC-3 was the most common carbapenemase we found (249; 20%); 96% of the KPC-3 was detected in *K. pneumoniae* belonging to clones sequence type (ST) 512 or ST258. In contrast, KPC-2, which comprised only 1.8% (23), was found in multiple species. Among OXA-48–like carbapenemases, which comprised 19% (254) of carbapenemases, 87% (221/254) were found in *K. pneumoniae*, 70% (155/221) of those in clones ST11, ST15, ST392, or ST307. 

**Figure 1:**
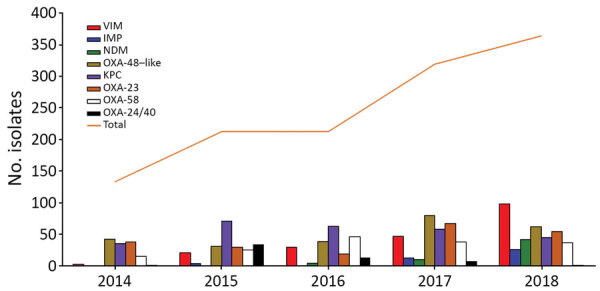
Distribution of carbapenemase types recovered in Andalusia, 2014–2018. IMP, imipenemase; KPC, *Klebsiella pneumoniae* carbapenemase; MBL, metallo-β-lactamases; NDM, New Delhi metallo-beta-lactamase; OXA, oxacillinases; VIM, Verona integron-encoded metallo-β-lactamase.

Over the course of the study period, we observed an increase in MBL carbapenemases, with a noteworthy increase in 2018 (Table 1; Figure 1). *Klebsiella* was most prevalent MBL, comprising 53% (157/294), almost equally distributed between *K. pneumoniae* and *K. oxytoca,* followed by *Pseudomonas aeruginosa* (20%, 60/294) and *Enterobacter cloacae* (14%, 42/294). The distribution of each MBL enzyme type varied significantly. We found 39% (77/197) of VIM in *K. oxytoca*, 80% (45/56) of NDM in *K. pneumoniae*, and 73% (30/41) of IMP in *P. aeruginosa*. OXA-23 (49%, 208/425) was the most common carbapenemase producer in *A. baumannii*, followed by OXA-58 (38%, 162/425), and OXA-24/40 (13%, 55/425). OXA-23 and OXA 24/40 isolates belonged predominantly to clone ST2. OXA-58 producers were detected in multiple clones. We summarize the distribution of carbapenemases among CPGNB in this study in Table 2. 

**Table 1:** Distribution of carbapenemase-producing gram-negative bacteria by year, Andalusia, Spain, 2014–2018

Carbapenemase	Bacteria	Year				
		2014	2015	2016	2017	2018
VIM	*Klebsiella pneumoniae*	0	0	9	9	15
	*K. oxytoca*	1	13	12	19	32
	*Escherichia coli*	0	2	1	3	2
	*Enterobacter cloacae*	0	4	4	10	15
	*Citrobacter freundii*	0	1	3	2	2
	*C. amalonaticus*	0	0	1	0	0
	*Pseudomonas aeruginosa*	1	0	0	0	29
	*P. putida*	0	0	0	3	3
	*Acinetobacter spp*	0	0	0	1	0
IMP	*K. pneumoniae*	0	0	0	0	2
	*E. coli*	0	0	0	0	1
	*E. cloacae*	0	0	0	2	5
	*C. freundii*	0	1	0	0	0
	*P. aeruginosa*	0	2	0	10	18
NDM	*K. pneumoniae*	0	0	1	7	37
	*E. coli*	0	0	1	1	3
	*E. cloacae*	0	0	2	0	0
	*Acinetobacter spp*	0	0	0	0	1
	*A. baumannii*	0	0	0	2	1
KPC	*K. pneumoniae*	35	71	61	54	38
	*K. oxytoca*	0	0	0	0	2
	*E. coli*	0	0	0	0	3
	*E. cloacae*	0	0	2	3	2
	*C. freundii*	0	0	0	1	0
OXA-48	*K. pneumoniae*	35	26	39	77	44
	*K. oxytoca*	0	0	0	1	3
	*E. coli*	6	3	0	1	3
	*E. cloacae*	1	1	0	0	5
	*C. freundii*	0	1	0	1	3
	*K. aerogenes*	0	0	0	0	4
OXA-23	*A. baumannii*	38	30	19	67	54
OXA-58	*A. baumannii*	15	25	46	38	36
OXA-24‎/40	*A. baumannii*	1	33	12	7	1
IMI	*E. cloacae*	0	0	0	0	1
IMP, imipenemase; KPC, *Klebsiella pneumoniae* carbapenemase; MBL, metallo-β-lactamases; NDM, New Delhi metallo-beta-lactamase; OXA, oxacillinases; VIM, Verona integron-encoded metallo-β-lactamase.						

**Table 2:** Distribution of carbapenemases by species, Andalusia, Spain, 2014–2018*

	Species										
	*K. p.*	*K. o.*	*E. coli*	*Enterobacter cloacae*	*C. f.*	*C. a.*	*Pseudomonas* *aeruginosa*	*P.* *putida*	*Acinetobacter* spp.	*A.* *baumannii*	*K. a.*
VIM-1	33	77	8	33	8	1	1	5	1	0	0
VIM-2	0	0	0	0	0	0	29	1	0	0	0
IMP-8	2	0	1	6	1	0	14	0	0	0	0
IMP-16	0	0	0	0	0	0	9	0	0	0	0
IMP-23	0	0	0	0	0	0	7	0	0	0	0
NDM-1	6	0	0	2	0	0	0	0	1	3	0
NDM-7	39	0	1	0	0	0	0	0	0	0	0
NDM-5	0	0	4	0	0	0	0	0	0	0	0
IMP-22	0	0	0	1	0	0	0	0	0	0	0
KPC-3	248	0	0	1	0	0	0	0	0	0	0
KPC-2	10	2	3	6	1	0	0	0	0	0	0
OXA-48	205	4	12	7	4	0	0	0	0	0	4
OXA-23	0	0	0	0	0	0	0	0	0	208	0
OXA-58	0	0	0	0	0	0	0	0	0	160	0
OXA-24‎/40	0	0	0	0	0	0	0	0	0	54	0
OXA-245	15	0	1	0	1	0	0	0	0	0	0
OXA-181	1	0	0	0	0	0	0	0	0	0	0
KPC-31	1	0	0	0	0	0	0	0	0	0	0
IMI-1	0	0	0	1	0	0	0	0	0	0	0
Total	560	83	30	57	15	1	60	6	2	425	4

*C. f., *Citrobacter freundii*; *C. a.*, *Citrobacter amalonaticus*; *E.*, *Escherichia*; *K. a.*, *Klebsiella aerogenes*; *K. o.*, *K. oxytoca*; *K. p.*, *Klebsiella pneumoniae*. IMP, imipenemase; KPC, *Klebsiella pneumoniae* carbapenemase; MBL, metallo-β-lactamases; NDM, New Delhi metallo-beta-lactamase; OXA, oxacillinases; VIM, Verona integron-encoded metallo-β-lactamase.

Geographically, carbapenemase producers were found in 32 hospitals distributed throughout 8 provinces of the region, with KPC detected in 26 hospitals, followed by OXA-48–like in 23 and MBL in 18 (Appendix Figure). Sequence type was available for 1,181 of 1,243 (95%) isolates. Some circulating high-risk clones were detected in *K. pneumoniae*, *E. cloacae*, *Escherichia coli*, *A. baumannii*, and *P. aeruginosa*. The combinations of species, carbapenemase variants, and sequence types are listed in the Appendix Table. 

**Figure 2:**
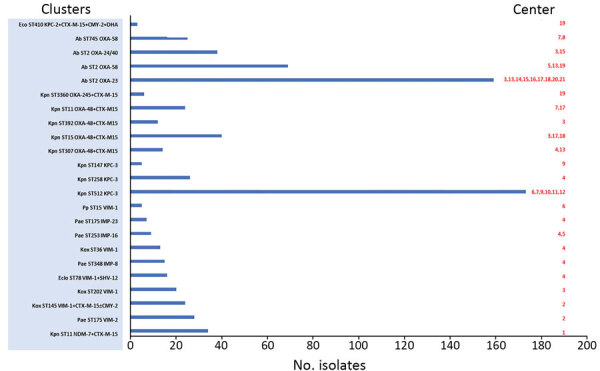
Characteristics of clusters of carbapenemase-producing gram negative bacteria, Andalusia, 2014–2018. Hospitals are identified by number: Almería ,1, 2, 5; Cádiz, 7, 8, 9; Córdoba, 6, 11, 12; Granada, 4, 13; Huelva, 20; Jaén, 10, 21; Málaga, 17, 18, 19; Sevilla, 3, 14, 15, 16. Ab, *Acinetobacter baumannii*; CTX-M, Cefotaximase-Munich; Eclo, *Enterobacter cloacae*; IMP, imipenemase; Kox, *Klebsiella oxytoca*; KPC, *Klebsiella pneumoniae* carbapenemase; Kpn, *Klebsiella pneumoniae*; MBL, metallo-β-lactamases; NDM, New Delhi metallo-beta-lactamase; OXA, oxacillinases; Pae, *Pseudomonas aeruginosa*; Pp, *Pseudomonas putida*; ST, sequence type; VIM, Verona integron-encoded metallo-β-lactamase.

Six isolates (0.5%) produced 2 types of carbapenemase: Five isolates combined VIM-1 with either OXA-48 (*K. oxytoca* ST206 and *E. cloacae* ST114), OXA-245 (*Citrobacter freundii* ST63), IMP-8 (*E. cloacae* ST242), or NDM-1 (*C. freundii* ST170). The remaining isolate was *E. coli* ST617 harboring NDM-5 and OXA-48. According to pulsed-field gel electrophoresis, 765 of 1,243 isolates (62%) were included in 23 clusters submitted from 22 hospitals (Figure 2).

## Conclusions

Our results provide an overview of the molecular epidemiology of CPGNB in Andalusia. The predominance of *K. pneumoniae* as a producer of all classes of carbapenemases is noteworthy, reflecting its role in disseminating carbapenemases in the region. KPC-3 and OXA-48 were the carbapenemases most frequently detected. KPC-3 was almost exclusively associated with *K. pneumoniae* and importantly with high-risk clones ST512 and ST258 considered endemic in other European countries (*8*–*10*). Like KPC-3, OXA-48 was predominantly found in *K. pneumoniae*, but it began appearing in other species in 2018, which raises concerns about the control of this carbapenemase in the region. In addition, the high-risk clones of OXA-48–producing *K. pneumoniae*, ST11, ST15, ST392, ST307, differed from those for KPC-3, showing that specific clones have a role in the dissemination of specific carbapenemases in this region. 

We found a significant temporal change in the epidemiology of MBL with a clear upward trend associated with several outbreaks in 2018. Findings from recent studies from Spain reflect the emergence of MBL in different CPGNB species (*4*,*11*,*12*). VIM was the MBL most frequently detected and, most of the VIM-producing bacteria were detected as part of clusters. IMP was almost exclusively found in *P. aeruginosa* isolates, showing the importance of this species in the dissemination of this carbapenemase; it was associated with different clusters in a single hospital and each variant was associated with a specific clone: IMP-8/ST348, IMP-23/ST175 and IMP-16/ST253. An increase in NDM was observed in 2018 associated with a large outbreak produced by *K. pneumoniae* ST11 NDM-7 in a single hospital.

In *A. baumanni*, OXA-23 and OXA-58 were the most common carbapenemases. ST2 was the predominant clone according to previous studies (*13*). A remarkable finding was the detection of *A. baumannii* ST85 harboring NDM-1 for the first time in Spain (*14*). The increase of carbapenemases in *E. coli* is a finding that could have epidemiologic significance because *E. coli* spread in the community more readily than other species and could begin to establish carbapenemases in the community. It is also remarkable that 7 of 23 clusters were detected in the same hospital, reflecting the burden of antimicrobial resistance in this hospital. This issue remains serious, and additional efforts for infection control should be made.

One limitation of this work is that, because submission of isolates is not mandatory, underreporting is likely. Nevertheless, we have evidence that most relevant isolates are routinely submitted. Furthermore, this study reinforces the importance of submitting all multidrug-resistant isolates to a reference laboratory because the information provided using molecular techniques makes the rapid detection of clusters and high-risk clones possible, which in turn, contributes to the success of infection prevention and control programs. 

In summary, our study shows dissemination and establishment of CPGNB in Andalusia, including high-risk clones of different species. These findings demonstrate the importance of continuing surveillance programs and epidemiologic studies to detect and investigate the spread of carbapenemase-producing bacteria, particularly in Andalusia, which is a region predisposed to the introduction of new lineages because of tourism and migration pathways. 

Appendix Additional information for carbapenemase-producing gram-negative bacteria in Andalusia, Spain, 2014–2018.
