# Supplementary material for: Carbapenemase-Producing Gram-Negative Bacteria in Andalusia, Spain, 2014–2018
Source: Emerg Infect Dis. 2020 Sep;26(9):2218–22. doi: 10.3201/eid2609.191772 (PMC7454067; doi:10.3201/eid2609.191772)
Supplement: Appendix — Additional information for carbapenemase-producing gram-negative bacteria in Andalusia, Spain, 2014–2018. [file 19-1772-Techapp-s1.pdf]

Article DOI: https://doi.org/10.3201/eid2609.191772

Carbapenemase-Producing Gram Negative Bacteria in Andalusia, Spain, 2014–2018

Appendix

Appendix Table. Distribution of carbapenemase and STs among the 1243 isolates included in the study

Species	Clone	Carbapenemase VIM -1	VIM -2	IMP -8	IMP -16	IMP -23	IMP -22	NDM -1	NDM -7	NDM -5	KPC -3	KPC -2	KPC -31	IMI -1	OXA -48	OXA -245	OXA -181	OXA -23	OXA -58	OXA -24/40	Total
Klebsiella pneumoniae	ST525	6	0	0	0	0	0	0	0	0	0	0	0	0	0	0	0	0	0	0	6
	ST111	1	0	0	0	0	0	0	0	0	0	0	0	0	0	0	0	0	0	0	1
	ST37	1	0	0	0	0	0	0	0	0	0	0	0	0	0	0	0	0	0	0	1
	ST307	1	0	0	0	0	0	4	3	0	1	3	0	0	22	0	0	0	0	0	34
	ST17	1	0	0	0	0	0	0	0	0	0	0	0	0	2	0	0	0	0	0	3
	ST22	0	0	0	0	0	0	0	0	0	0	0	0	0	1	0	0	0	0	0	1
	ST395	0	0	0	0	0	0	2	0	0	0	0	0	0	0	0	0	0	0	0	2
	ST348	1	0	0	0	0	0	0	0	0	0	0	0	0	0	0	0	0	0	0	1
	ST36	1	0	0	0	0	0	0	0	0	0	0	0	0	0	0	0	0	0	0	1
	ST25	1	0	0	0	0	0	0	0	0	0	0	0	0	0	0	0	0	0	0	1
	ST514	1	0	0	0	0	0	0	0	0	0	0	0	0	0	0	0	0	0	0	1
	ST2856	1	0	0	0	0	0	0	0	0	0	0	0	0	3	0	0	0	0	0	4
	ST15	11	0	0	0	0	0	0	0	0	0	0	0	0	66	3	0	0	0	0	80
	ST128	1	0	0	0	0	0	0	0	0	0	0	0	0	0	0	0	0	0	0	1
	ST11	1	0	0	0	0	0	0	35	0	0	0	0	0	41	4	0	0	0	0	81
	ST392	0	0	1	0	0	0	0	0	0	0	0	0	0	19	0	0	0	0	0	20
	ST202	2	0	0	0	0	0	0	0	0	0	0	0	0	0	0	0	0	0	0	2
	ST13	1	0	0	0	0	0	0	0	0	0	0	0	0	5	0	0	0	0	0	6
	ST321	0	0	1	0	0	0	0	0	0	0	0	0	0	0	0	0	0	0	0	1
	ST152	0	0	0	0	0	0	0	1	0	0	0	0	0	0	0	0	0	0	0	1
	ST1079	1	0	0	0	0	0	0	0	0	0	0	0	0	0	0	0	0	0	0	1
	ST1308	1	0	0	0	0	0	0	0	0	0	0	0	0	0	0	0	0	0	0	1
	ST464	0	0	0	0	0	0	0	0	0	0	0	0	0	1	0	0	0	0	0	1
	ST512	0	0	0	0	0	0	0	0	0	209	0	1	0	0	0	0	0	0	0	210
	ST258	0	0	0	0	0	0	0	0	0	31	0	0	0	0	0	0	0	0	0	31
	ST753	0	0	0	0	0	0	0	0	0	0	0	0	0	1	0	0	0	0	0	1
	ST147	0	0	0	0	0	0	0	0	0	5	1	0	0	8	0	0	0	0	0	14
	ST836	0	0	0	0	0	0	0	0	0	0	0	0	0	0	0	1	0	0	0	1
	ST899	0	0	0	0	0	0	0	0	0	0	0	0	0	1	0	0	0	0	0	1
	ST1	0	0	0	0	0	0	0	0	0	0	0	0	0	3	2	0	0	0	0	5

Species	Clone	Carbapenemase																			
		VIM -1	VIM -2	IMP -8	IMP -16	IMP -23	IMP -22	NDM -1	NDM -7	NDM -5	KPC -3	KPC -2	KPC -31	IMI -1	OXA -48	OXA -245	OXA -181	OXA -23	OXA -58	OXA -24/40	Total
	ST431	0	0	0	0	0	0	0	0	0	0	0	0	0	1	0	0	0	0	0	1
	ST251	0	0	0	0	0	0	0	0	0	0	0	0	0	0	1	0	0	0	0	1
	ST974	0	0	0	0	0	0	0	0	0	0	0	0	0	1	0	0	0	0	0	1
	ST405	0	0	0	0	0	0	0	0	0	0	0	0	0	0	1	0	0	0	0	1
	ST391	0	0	0	0	0	0	0	0	0	0	0	0	0	1	0	0	0	0	0	1
	ST225	0	0	0	0	0	0	0	0	0	0	0	0	0	1	0	0	0	0	0	1
	ST34	0	0	0	0	0	0	0	0	0	0	1	0	0	0	0	0	0	0	0	1
	ST16	0	0	0	0	0	0	0	0	0	0	0	0	0	1	0	0	0	0	0	1
	ST1963	0	0	0	0	0	0	0	0	0	0	1	0	0	0	0	0	0	0	0	1
	ST981	0	0	0	0	0	0	0	0	0	0	0	0	0	1	0	0	0	0	0	1
	ST340	0	0	0	0	0	0	0	0	0	0	0	0	0	0	1	0	0	0	0	1
	ST1732	0	0	0	0	0	0	0	0	0	0	0	0	0	0	1	0	0	0	0	1
	ST2262	0	0	0	0	0	0	0	0	0	0	0	0	0	2	0	0	0	0	0	2
	ST3358	0	0	0	0	0	0	0	0	0	0	3	0	0	0	0	0	0	0	0	3
	ST3362	0	0	0	0	0	0	0	0	0	0	0	0	0	9	0	0	0	0	0	9
	ST1562	0	0	0	0	0	0	0	0	0	0	1	0	0	0	0	0	0	0	0	1
	ST846	0	0	0	0	0	0	0	0	0	0	0	0	0	1	0	0	0	0	0	1
	ST3360	0	0	0	0	0	0	0	0	0	0	0	0	0	0	2	0	0	0	0	2
	ST442	0	0	0	0	0	0	0	0	0	0	0	0	0	5	0	0	0	0	0	5
	ST716	0	0	0	0	0	0	0	0	0	0	0	0	0	1	0	0	0	0	0	1
	NA	0	0	0	0	0	0	0	0	0	2	0	0	0	8	0	0	0	0	0	10
K. oxytoca	ST145	24	0	0	0	0	0	0	0	0	0	0	0	0	1	0	0	0	0	0	25
	ST36	14	0	0	0	0	0	0	0	0	0	0	0	0	0	0	0	0	0	0	14
	ST170	0	0	0	0	0	0	0	0	0	0	1	0	0	0	0	0	0	0	0	1
	ST27	5	0	0	0	0	0	0	0	0	0	0	0	0	0	0	0	0	0	0	5
	ST213	1	0	0	0	0	0	0	0	0	0	0	0	0	0	0	0	0	0	0	1
	ST202	22	0	0	0	0	0	0	0	0	0	0	0	0	2	0	0	0	0	0	24
	ST206	1	0	0	0	0	0	0	0	0	0	0	0	0	0	0	0	0	0	0	1
	ST82	1	0	0	0	0	0	0	0	0	0	0	0	0	0	0	0	0	0	0	1
	ST214	0	0	0	0	0	0	0	0	0	0	0	0	0	1	0	0	0	0	0	1
	ST95	0	0	0	0	0	0	0	0	0	0	1	0	0	0	0	0	0	0	0	1
	NA	9	0	0	0	0	0	0	0	0	0	0	0	0	0	0	0	0	0	0	9
K. aerogenes	ST93	0	0	0	0	0	0	0	0	0	0	0	0	0	3	0	0	0	0	0	3
	ST153	0	0	0	0	0	0	0	0	0	0	0	0	0	1	0	0	0	0	0	1
Escherichia coli	ST1193	0	0	0	0	0	0	0	0	1	0	0	0	0	0	0	0	0	0	0	1
	ST145	1	0	0	0	0	0	0	0	0	0	0	0	0	0	0	0	0	0	0	1
	ST348	0	0	1	0	0	0	0	0	0	0	0	0	0	0	0	0	0	0	0	1
	ST8327	1	0	0	0	0	0	0	0	0	0	0	0	0	0	0	0	0	0	0	1
	ST141	0	0	0	0	0	0	0	1	0	0	0	0	0	0	0	0	0	0	0	1
	ST88	1	0	0	0	0	0	0	0	0	0	0	0	0	0	0	0	0	0	0	1
	ST410	0	0	0	0	0	0	0	0	2	0	3	0	0	0	0	0	0	0	0	5
	ST617	0	0	0	0	0	0	0	0	1	0	0	0	0	0	0	0	0	0	0	1
	ST69	0	0	0	0	0	0	0	0	0	0	0	0	0	1	0	0	0	0	0	1
	ST1340	0	0	0	0	0	0	0	0	0	0	0	0	0	1	0	0	0	0	0	1
	ST12	0	0	0	0	0	0	0	0	0	0	0	0	0	1	0	0	0	0	0	1
	NA	5	0	0	0	0	0	0	0	0	0	0	0	0	9	1	0	0	0	0	15
	ST78	19	0	2	0	0	0	0	0	0	0	0	0	0	0	0	0	0	0	0	21

		Carbapenemase																			
Species	Clone	VIM -1	VIM -2	IMP -8	IMP -16	IMP -23	IMP -22	NDM -1	NDM -7	NDM -5	KPC -3	KPC -2	KPC -31	IMI -1	OXA -48	OXA -245	OXA -181	OXA -23	OXA -58	OXA -24/40	Total
Enterobacter cloacae	ST111	2	0	0	0	0	0	0	0	0	0	0	0	0	0	0	0	0	0	0	2
	ST932	0	0	0	0	0	0	2	0	0	0	0	0	0	0	0	0	0	0	0	2
	ST90	2	0	0	0	0	0	0	0	0	0	1	0	0	0	0	0	0	0	0	3
	ST114	2	0	1	0	0	0	0	0	0	0	0	0	0	2	0	0	0	0	0	5
	ST528	1	0	0	0	0	0	0	0	0	0	0	0	0	0	0	0	0	0	0	1
	ST96	0	0	0	0	0	1	0	0	0	1	0	0	0	0	0	0	0	0	0	2
	ST50	1	0	0	0	0	0	0	0	0	0	0	0	0	0	0	0	0	0	0	1
	ST413	0	0	1	0	0	0	0	0	0	0	0	0	0	0	0	0	0	0	0	1
	ST50	1	0	0	0	0	0	0	0	0	0	0	0	0	0	0	0	0	0	0	1
	ST242	3	0	0	0	0	0	0	0	0	0	0	0	0	0	0	0	0	0	0	3
	ST24	0	0	2	0	0	0	0	0	0	0	0	0	0	0	0	0	0	0	0	2
	ST110	0	0	0	0	0	0	0	0	0	0	1	0	0	0	0	0	0	0	0	1
	ST991	0	0	0	0	0	0	0	0	0	0	1	0	0	0	0	0	0	0	0	1
	ST74	0	0	0	0	0	0	0	0	0	0	1	0	0	0	0	0	0	0	0	1
	ST108	0	0	0	0	0	0	0	0	0	0	0	0	0	1	0	0	0	0	0	1
	ST32	0	0	0	0	0	0	0	0	0	0	1	0	0	0	0	0	0	0	0	1
	ST1107	0	0	0	0	0	0	0	0	0	0	0	0	0	1	0	0	0	0	0	1
	ST113	0	0	0	0	0	0	0	0	0	0	0	0	0	1	0	0	0	0	0	1
	ST901	0	0	0	0	0	0	0	0	0	0	0	0	1	0	0	0	0	0	0	1
	NA	2	0	0	0	0	0	0	0	0	0	1	0	0	2	0	0	0	0	0	5
Citrobacter freundii	ST8	1	0	1	0	0	0	0	0	0	0	0	0	0	0	0	0	0	0	0	2
	ST22	1	0	0	0	0	0	0	0	0	0	0	0	0	0	0	0	0	0	0	1
	ST44	1	0	0	0	0	0	0	0	0	0	0	0	0	0	0	0	0	0	0	1
	ST63	1	0	0	0	0	0	0	0	0	0	0	0	0	0	0	0	0	0	0	1
	ST170	1	0	0	0	0	0	0	0	0	0	0	0	0	0	0	0	0	0	0	1
	ST408	1	0	0	0	0	0	0	0	0	0	0	0	0	0	0	0	0	0	0	1
	ST410	1	0	0	0	0	0	0	0	0	0	0	0	0	0	0	0	0	0	0	1
	ST112	0	0	0	0	0	0	0	0	0	0	0	0	0	2	0	0	0	0	0	2
	ST261	0	0	0	0	0	0	0	0	0	0	0	0	0	1	0	0	0	0	0	1
	ST425	0	0	0	0	0	0	0	0	0	0	0	0	0	1	0	0	0	0	0	1
	NA	1	0	0	0	0	0	0	0	0	0	1	0	0	0	1	0	0	0	0	3
C. amalonatycus	NA	1	0	0	0	0	0	0	0	0	0	0	0	0	0	0	0	0	0	0	1
Pseudomonas aeruginosa	ST253	0	0	0	9	0	0	0	0	0	0	0	0	0	0	0	0	0	0	0	9
	ST348	0	0	14	0	0	0	0	0	0	0	0	0	0	0	0	0	0	0	0	14
	ST175	0	28	0	0	7	0	0	0	0	0	0	0	0	0	0	0	0	0	0	35
	ST308	0	1	0	0	0	0	0	0	0	0	0	0	0	0	0	0	0	0	0	1
	NA	1	0	0	0	0	0	0	0	0	0	0	0	0	0	0	0	0	0	0	1
P. putida	ST15	5	0	0	0	0	0	0	0	0	0	0	0	0	0	0	0	0	0	0	5
	NA	0	1	0	0	0	0	0	0	0	0	0	0	0	0	0	0	0	0	0	1
Acinetobacter baumannii	ST307	0	0	0	0	0	0	0	0	0	0	0	0	0	0	0	0	15	0	0	15
	ST85	0	0	0	0	0	0	3	0	0	0	0	0	0	0	0	0	8	0	0	11
	ST2	0	0	0	0	0	0	0	0	0	0	0	0	0	0	0	0	170	83	49	302
	ST98	0	0	0	0	0	0	0	0	0	0	0	0	0	0	0	0	0	21	0	21
	ST829	0	0	0	0	0	0	0	0	0	0	0	0	0	0	0	0	1	0	0	1
	ST745	0	0	0	0	0	0	0	0	0	0	0	0	0	0	0	0	1	55	0	56
	ST32	0	0	0	0	0	0	0	0	0	0	0	0	0	0	0	0	0	0	1	1
	ST164	0	0	0	0	0	0	0	0	0	0	0	0	0	0	0	0	2	0	0	2

		Carbapenemase																			
Species	Clone	VIM -1	VIM -2	IMP -8	IMP -16	IMP -23	IMP -22	NDM -1	NDM -7	NDM -5	KPC -3	KPC -2	KPC -31	IMI -1	OXA -48	OXA -245	OXA -181	OXA -23	OXA -58	OXA -24/40	Total
	NA	0	0	0	0	0	0	0	0	0	0	0	0	0	0	0	0	11	1	4	16
Acinetobacter spp.	ST119	0	0	0	0	0	0	1	0	0	0	0	0	0	0	0	0	0	0	0	1
	NA	1	0	0	0	0	0	0	0	0	0	0	0	0	0	0	0	0	0	0	1
Total		167	30	24	9	7	1	12	40	4	249	22	1	1	236	17	1	208	160	54	1243

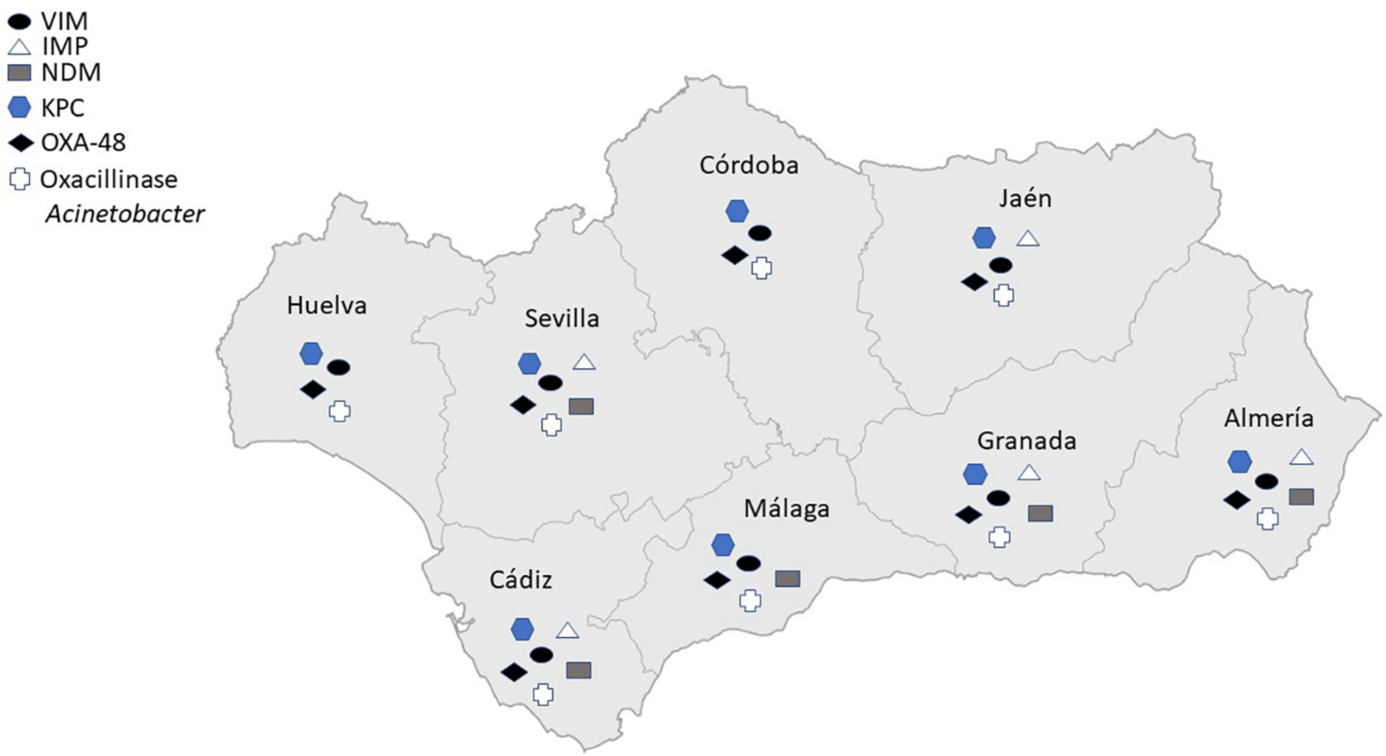

Appendix Figure. Geographic distribution of carbapenemase types.
